# Relevance of Type II Endoleak After Endovascular Repair of Ruptured Abdominal Aortic Aneurysms: A Retrospective Single-Center Cohort Study

**DOI:** 10.1177/15266028221086476

**Published:** 2022-03-30

**Authors:** Anna-Leonie Menges, Lorenz Meuli, Philip Dueppers, Kerstin Stoklasa, Reinhard Kopp, Benedikt Reutersberg, Alexander Zimmermann

**Affiliations:** 1 Department of Vascular Surgery, University Hospital Zurich, Zurich, Switzerland

**Keywords:** endoleak, aortic aneurysm rupture, EVAR, T2EL, type II endoleak

## Abstract

**Introduction:**

Endovascular aortic repair (EVAR) is widely used as an alternative to open repair in elective and even in emergent cases of ruptured abdominal aortic aneurysm (rAAA). One of the most frequent complications after EVAR is type II endoleak (T2EL). In elective therapy, evidence-based therapeutic recommendations for T2EL are limited. Completely unclear is the role of T2EL after EVAR for rAAA (rEVAR). This study aims to investigate the significance of T2ELs after rEVAR.

**Patients and methods:**

This is a retrospective single-center data analysis of all patients who underwent rEVAR between January 2010 and December 2020 with primary T2EL. The outcome criteria were overall and T2EL-related mortality and reintervention rate as well as development of aneurysm diameter over follow-up (FU).

**Results:**

During the study period between January 2010 and December 2020, 35 (25%) out of 138 patients with rEVAR presented a primary postoperative T2EL (age 74±11 years, 34 males). At rupture, mean aneurysm diameter was 73±12 mm. Follow-up was 26 (0–172) months. The reintervention-free survival was 69% (95% confidence interval [CI]: 55%–86%) at 30 days, 58% (95% CI: 43%–78%) at 1 year, and 52% (95% CI: 36%–75%) at 3 years. In 40% (n=14), T2ELs resolved spontaneously within a median time of 3.4 (0.03–85.6) months. The overall and T2EL reintervention rates were 43% (n=15) and 9% (n=3), respectively. Within 30 days, 11 patients (31%) required reintervention, of which 2 were T2EL related. Aneurysm sac growth by ≥5 mm was seen in 3 patients (9%), and aneurysm shrinkage rate was significantly higher in sealed T2EL group (86% vs 5%, p<0.0001). The overall survival was 85% (95% CI: 74%–98%) at 30 days, 75% (95% CI: 61%–92%) at 1 year, and 67% (95% CI: 51%–87%) at 3 years. Six deaths were aneurysm related, while 1 was T2EL related within the first 30 days due to persistent hemorrhage. During FU, one more patient died due to a T2EL-related secondary rupture (T2EL-related mortality, 5.7%, n=2). Multivariable analysis revealed that arterial hypertension was associated with an increased risk for reintervention (hazard ratio [HR]: 27.8, 95% CI: 1.48–521, p=0.026) and age was associated with an increased risk for mortality (HR 1.14, 95% CI: 1.04–1.26, p=0.005).

**Conclusion:**

T2ELs after rEVAR showed a benign course in most cases. In the short term, the possibility of persistent bleeding should be considered. In the mid term, a consequent FU protocol is required to detect known late complications after EVAR at an early stage and to prevent secondary rupture and death.

## Introduction

Abdominal aortic aneurysms (AAA) have an age-related prevalence of 2% to 11%. It is a leading cause of death in men above 65 years if not treated at a threshold diameter of 55 mm.^1^ Due to its low invasiveness and morbidity, endovascular aortic repair (EVAR) as an alternative to open repair has become the therapy of choice in several vascular centers around the world in recent years.^2,3^ Therefore, it is used nowadays in up to 80% of elective cases and up to 60% of ruptured cases.^4^ However, the occurrence of endoleak remains a problem that has not been solved conclusively. Endoleaks are the most frequent reason for reintervention after elective EVAR.^5,6^ While the need and therapy strategy are clear for type I endoleak (T1EL) and type III endoleak (T3EL), there is still a lack of evidence how to treat type II endoleak (T2EL).^7^

The occurrence of a T2EL after elective EVAR is described in 8% to 44% of cases.^8,9^ More than 30% of T2EL disappear spontaneously.^10^ Over the last few years, there has been increasing evidence that the risk of secondary rupture due to an isolated T2EL is low overall, despite endovascular treatment of aortic aneurysm could lead to atrophy of the aortic wall.^11,12^

However, if a persistent T2EL leads to a progressive increase in size of aneurysm sac, treatment is recommended to prevent more severe complications such as the occurrence of T1EL.^3,13^ If the diameter remains stable, a conservative strategy with close follow-up (FU) is currently recommended.

While a consensus has emerged for the treatment of T2EL after elective EVAR, the development, treatment, and natural history of T2EL after ruptured abdominal aortic aneurysm (rAAA) are hardly described. There is only one report of 16 patients with T2EL after rAAA, demonstrating their benign character.^14^ However, some case reports describe a life-threatening persistent hemorrhage.^15–17^

Due to the very limited data available, the aim of this study was to evaluate the significance of a T2EL during FU of a rAAA treated with an EVAR in the short term and mid term.

## Patients and Methods

### Patients

In a retrospective data analysis, all patients that presented with rAAA between January 2010 and December 2020 were screened for eligibility. An AAA was defined as ruptured if the primary computed tomography (CT) scan showed a hematoma outside the aortic wall and in relation to the AAA. Patients were included if they were treated with EVAR and a T2EL was detected at the immediate postoperative computed tomographic angiogram (CTA).

Exclusion criteria were conservative, open-operative therapy, and proximal sealing zone above the renal arteries (eg, parallel grafts, physician-modified stent grafts).

Data acquisition was in accordance with the Declaration of Helsinki and either a written informed consent was obtained from all patients or their enrolment was provided by the Federal Human Research Act.

The Regional Review Board provided ethical approval (Nr. 2020-02820).

### Therapy and Follow up Protocol

All patients with CT graphically confirmed rAAA were evaluated for the feasibility of endovascular therapy. The stent graft size was selected based on CT graphic measurements in an interdisciplinary consensus of the vascular surgeons and interventional radiologists on duty. Immediately after EVAR a postoperative CTA was conducted to detect relevant endoleaks and to prove the correct positioning of the stent graft. After EVAR for rAAA (rEVAR), patients were monitored in the intermediate or intensive care unit depending on cardiopulmonary stability for at least 2 to 3 days.

Follow-up was performed using CTA to detect a persisting or new endoleak as well as to determine the maximum aortic aneurysm diameter after 4 to 6 weeks and 6 to 12 months postoperatively. Afterward, CTAs have been performed annually.

For this retrospective observational cohort study, FU information was included up to the prespecified study’s end date January 31, 2021. Completeness of FU information was quantified using the follow-up index (FUI).^18^

### Data Collection

Patients were identified in the institutional clinical information system (KISIM 5.1.0.3; CISTEC AG, Zurich). Baseline clinical data included gender, age, comorbidities, and antiplatelet or anticoagulation therapy. Perioperative and postoperative outcomes were evaluated by assessing mortality, postoperative complications (cardiac, respiratory, renal, gastrointestinal, wound healing, puncture site complications), persistence or disappearance of endoleaks, aneurysm diameter, and rate of reintervention. If required, the latest data were transferred from external hospitals. The federal “Unique Person Identification” registry was used for survival data.

Preoperative and postoperative CTAs were analyzed using a 3-dimensional workstation (XERO Viewer 8.1.2, Agfa HealthCare N.V., Mortsel).

Description of proximal neck quality was performed by measuring length, diameter, and angulation in CTA. Since Medtronic Endurant and GORE Excluder AAA were used in almost 95% of the cases, their instruction for use (IFU) was used as reference (neck diameter 19–32 mm, neck length >15 mm, and angulation ≤60°).

Endoleak and morphological analysis was independently performed by the first and senior author and inconsistencies were discussed before reaching an agreement.

### Outcome Criteria

Outcome criteria were overall and T2EL-related reintervention rate, including reintervention-free survival as well as overall, aneurysm-related and T2EL-related mortality rate. Further outcome criteria were development of aneurysm diameter and sealing rate of T2EL.

Aneurysm-related mortality was defined as any death due to complications in the postoperative hospital course or any death related to the aneurysm or graft at any time during following up. T2EL-related mortality was defined as death resulting from T2EL like persistent hemorrhage or secondary rupture related to T2EL.

Multivariable analysis on baseline characteristics was performed to assess their association with the risk for reintervention and mortality.

### Statistical Analysis

Overall survival and reintervention-free survival were assessed with mortality as a competing risk using a multivariable Cox proportional hazard model including baseline characteristics, comorbidities, and aneurysm morphology as potential confounders. The proportional hazard assumption was tested and confirmed for each of the variables and the overall model using scaled Schoenfeld residuals. Kaplan-Meier estimators were used for estimation of overall survival and the corresponding 95% confidence intervals (CIs).

Time-to-event analyses were performed with RStudio, version 3.6.3, R Core Team (2020). R Foundation is used for statistical computing, Vienna, Austria, https://www.R-project.org/, on macOS version 11.4.

Categorical variables are presented with counts and percentages and compared using chi-square test. Continuous variables are summarized by means and standard deviation if normally distributed or by median and interquartile range (IQR) if skewed. Continuous variables were compared using *t* test or Mann-Whitney *U* test, respectively. Statistical analyses were performed using Med-Calc© (MedCalc Software, Mariakerke, https://www.medcalc.org/calc/comparison_of_proportions.php; Version 20.013; accessed September 26, 2021), with an alpha level of 5%.

## Results

### Patients

**Figure 1.**
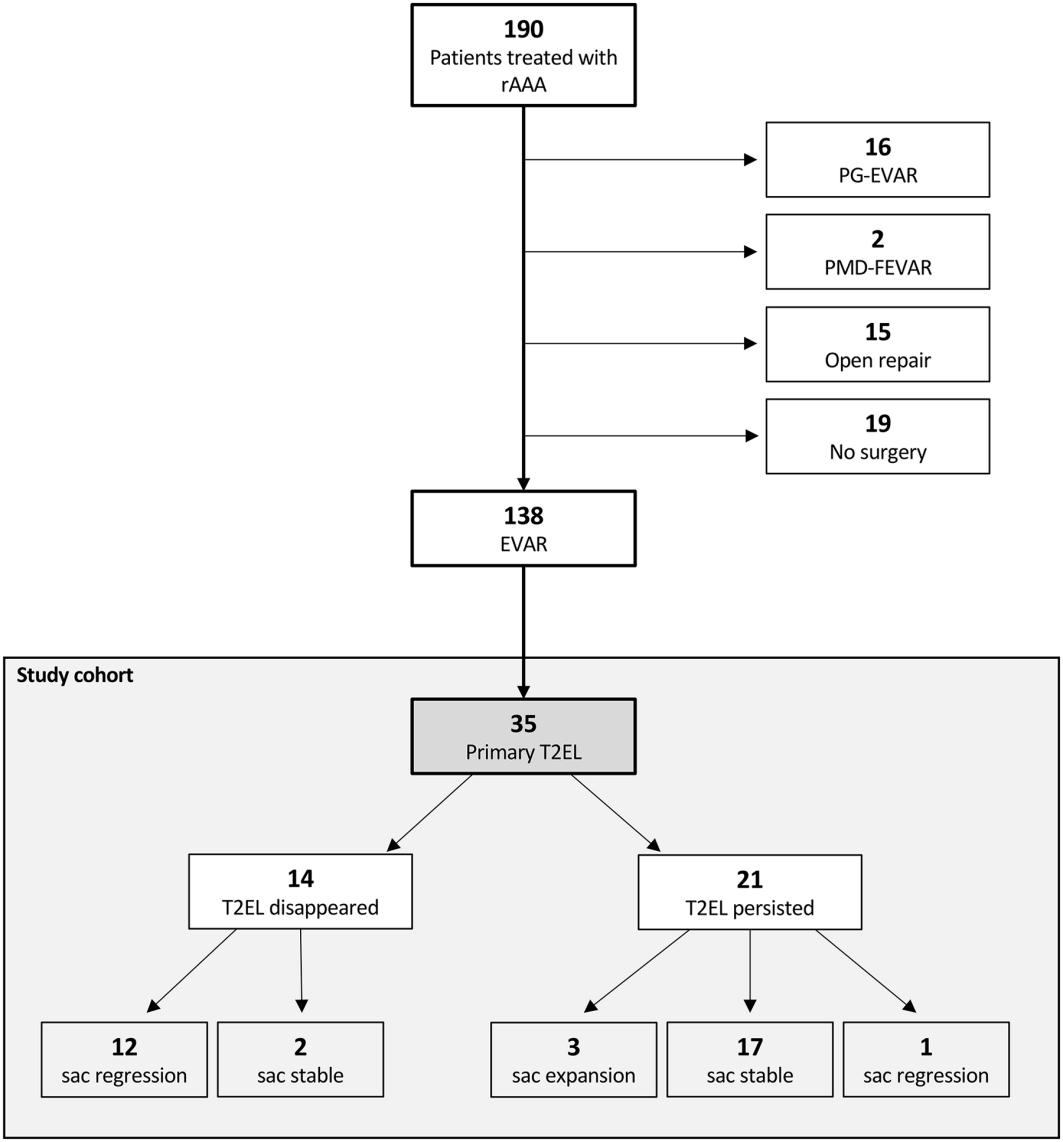
Flowchart of patients treated for ruptured abdominal aortic aneurysm between January 2010 and December 2020 and the development of type II endoleak (white) in the study cohort (gray). rAAA, ruptured abdominal aortic aneurysm; EVAR, endovascular aortic repair; PG-EVAR, Parallel grafts EVAR; PMD-FEVAR, physician-modified fenestrated EVAR; T2EL, type II endoleak.

Between January 2010 and December 2020, a total of 190 patients with a rAAA were treated in our hospital. In total, 19 patients received conservative therapy (best supportive care), 15 underwent surgical repair, 18 received a more complex endovascular aortic procedure (parallel graft EVAR: n=16 or physician-modified EVAR: n=2), and 138 received a standard EVAR. Intraoperative or postoperative imaging revealed 4 T1EL (3%) and no T3EL. T2EL was evident in 35 cases (25%) on the first postinterventional imaging (Figure 1).

The mean diameter of the AAA of these 35 patients at rupture was 73±12 mm, the mean age was 74±11 years, and 34 (97%) patients were male. The median FU time was 21 (IQR: 2–40) months with good availability of FU information (median FUI: 0.83, IQR: 0.49–1.0). Aortic endoprosthesis was implanted within the IFU in 54% (n=19) of patients.

Detailed patient characteristics and morphological parameters are summarized in Table 1.

**Table 1.** Baseline Characteristics.

	Total (n=35)
Demographics	
Age, years (SD)	74 (11)
Male sex, n (%)	34 (97)
Comorbidities	
Arterial hypertension, n (%)	31 (89)
Ever smoker, n (%)	15 (43)
Statin use, n (%)	17 (49)
Diabetes mellitus, n (%)	5 (14)
Coronary artery disease, n (%)	10 (29)
Chronic kidney disease, n (%)	16 (46)
COPD, n (%)	9 (26)
Antithrombotic therapy	
APT, n (%)	26 (74)
Dual APT, n (%)	2 (6)
Anticoagulation, n (%)	9 (26)
Anticoagulation and APT, n (%)	3 (9)
Morphology	
Aneurysm diameter at rupture, mm (SD)	73 (12)
Median neck diameter at rupture, mm (IQR)	20 (18–24.5)
Neck length >10 mm, n (%)	29 (83)
Neck angulation ≤60°, n (%)	32 (91)
Compliant with instructions for use, n (%)	19 (54)
Graft	
Cook Zenith, n (%)	1 (3)
GORE Excluder, n (%)	9 (26)
Medtronic Endurant, n (%)	24 (67)
Other, n (%)	1 (3)

Data were complete if not stated otherwise. Counts are presented with percentage (%), continuous variables and summarized by mean and SD if normally distributed or median and IQR if skewed.

Abbreviations: APT, antiplatelet therapy; COPD, chronic obstructive pulmonary disease; IQR, interquartile range; SD, standard deviation.

### Reinterventions

**Figure 2.**
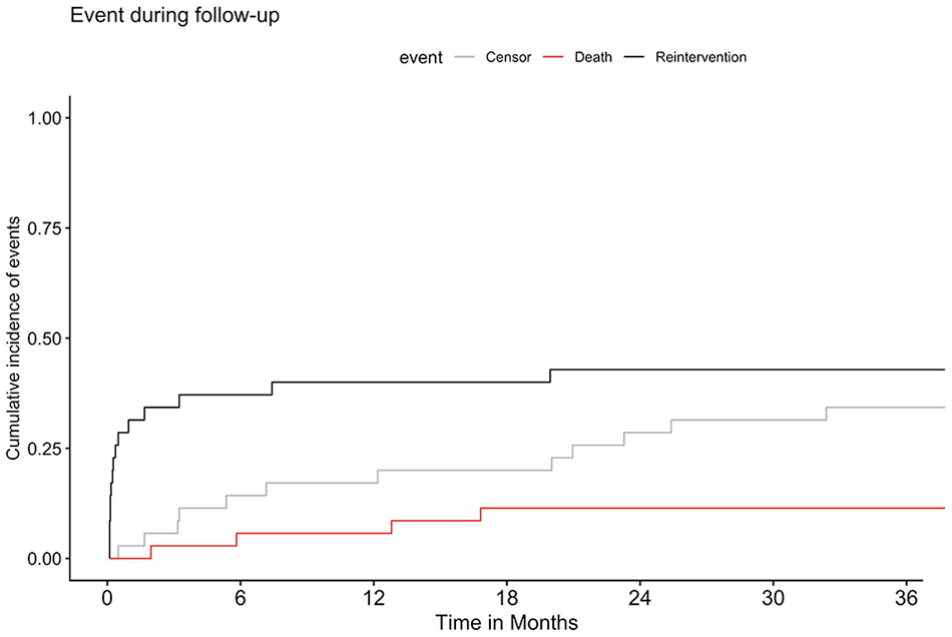
Competing risk analysis of reintervention-free survival over a course of 3 years after endovascular treatment for ruptured abdominal aneurysm. Median (line) and 95% confidence intervals (area).

The reintervention-free survival was 69% (95% CI: 55–86%) at 30 days, 58% (95% CI: 43–78%) at 1 year, and 52% (95% CI: 36–75%) at 3 years (Figure 2).

A total of 15 patients (43%) required reintervention during the FU period including 11 reinterventions (31%) within the first 30 days (Table 2).

**Table 2.** Thirty-Day and Overall Reintervention.

Indication	Procedure	Total (n=35) n (%)
**Reintervention <30 days**		11 (31.4)
T1EL		4 (11.4)
	Endoanchor	3 (8.6)
	Postdilatation	1 (2.9)
T2EL	Laparotomy/ligature of lumbar arteries	2 (5.7)^a^
T3EL	—	0 (0)
Short distal attachment	Distal extension	2 (5.7)
Abdominal compartment syndrome	Open abdomen treatment	3 (8.6)
Colon ischemia	Hemicolectomy	1 (2.9)
**Reintervention >30 days**		4 (11.4)
T1EL	Endoanchor + coiling	1 (2.9)
T2EL	Iliolumbar coiling	1 (2.9)
T3EL	—	0 (0)
Shortening proximal neck	Endoanchor + coiling	1 (2.9)
Endograft infect by mycotic aneurysm	Replacement with silver prosthesis	1 (2.9)
**Overall reintervention**		15 (42.9)
**Overall T2EL-related reintervention**		3 (8.6)

Results are given as numbers (%). Italic indicates details

Abbreviations: T1EL, type I endoleak; T2EL, type II endoleak; T3EL, type III endoleak.

a One patient died because the hemorrhage could not be stopped.

The overall reintervention rate for T2EL was 9% (n=3), whereby the 30 day T2EL reintervention rate was 6% (n=2). Both patients showed persistent bleeding via the lumbar arteries with hemodynamic instability and abdominal compartment syndrome (ACS) postoperatively. One of these patients was treated successfully with open ligation of the feeder vessels and open abdomen treatment. The other patient died on day 1 during reintervention because the hemorrhage could not be stopped.

Four early T1ELs were treated by proximal fixation with endoanchors (n=3) or dilatation of the proximal neck (n=1) immediately after operation.

The other 5 reinterventions within the first 30 days were explorative laparotomy with open abdomen treatment because of ACS (n=3), hemicolectomy because of colon ischemia (n=1), and distal extension of the endoprosthesis leg because of short distal attachment with impending risk for T1EL (n=1).

One T2EL was treated during the FU by iliolumbar coiling due to aneurysm sac increase 7.5 months after rupture. One T2EL-induced T1EL was treated successfully by proximal fixation with endoanchor and simultaneous coil embolization of the T2EL after 76 months. To prevent another T1EL due to shortening of the proximal neck, one further reintervention with endoanchor and simultaneous coil embolization was performed.

In another case, secondary open aortic replacement and removal of the stent graft was performed for mycotic aneurysm after 3 months (Table 2).

The multivariable Cox model for reintervention-free survival with mortality as a competing risk showed that the diagnosis of arterial hypertension was associated with an increased risk for reintervention (hazard ratio [HR]: 27.8, 95% CI: 1.48–521, p=0.026; Table 3). The other variables, especially neck diameter, neck length, and neck angulation, were not associated with freedom from reintervention. Of note, the CIs were inflated due to the low number of events and the small sample.

**Table 3.** Multivariable Cox Models on Overall Survival and Reintervention-Free Survival.

Overall survival					
Characteristic	Survivors (n=22)	Deceased (n=13)	HR	95% CI	p-Value
Age, years	**70.8 (10.1)**	**79.0 (9.5)**	**1.14**	**1.04–1.26**	**0.005**
Female sex	0	1 (8%)	1.23	0.10–14.7	0.871
Aneurysm diameter, mm	73.2 (10.9)	71.6 (14.6)	0.98	0.92–1.04	0.451
Coronary artery disease	8 (36%)	2 (15%)	0.56	0.11–2.94	0.491
COPD	5 (23%)	4 (31%)	1.83	0.18–18.8	0.612
Statin use	11 (50%)	6 (46%)	0.40	0.07–2.27	0.302
Arterial hypertension	19 (86%)	12 (92%)	15.3	0.31–762	0.172
Ever smoker	9 (41%)	6 (46%)	0.52	0.05–5.03	0.576
Chronic kidney disease	8 (36%)	2 (15%)	0.78	0.16–3.74	0.761
Diabetes mellitus	3 (14%)	2 (15%)	5.33	0.47–60.1	0.176
Reintervention-free survival					
Characteristic	No reintervention	Reintervention	HR	95% CI	p-Value
Age, years	73.5 (10.8)	74.3 (10.6)	1.05	0.98–1.14	0.167
Female sex	1 (5%)	0 (0%)	7.73	0.00–Inf	0.999
Aneurysm diameter, mm	75 (13)	69 (10)	0.98	0.92–1.05	0.612
Coronary artery disease	6 (30%)	4 (27%)	5.83	0.69–49.2	0.105
COPD	6 (30%)	3 (20%)	0.43	0.04–4.43	0.479
Statin use	10 (50%)	7 (47%)	0.19	0.03–1.26	0.085
Arterial hypertension	**18 (90%)**	**13 (87%)**	**27.8**	**1.48–521**	**0.026**
Ever smoker	9 (45%)	6 (40%)	2.61	0.34–20.2	0.358
Chronic kidney disease	6 (30%)	4 (27%)	0.59	0.12–3.04	0.532
Diabetes mellitus	4 (20%)	1 (7%)	6.66	0.43–103	0.175
Inside IFU	11 (55%)	8 (53%)	0.35	0.05–2.28	0.270
Neck diameter, mm	22 (18–24)	19 (18–23)	0.90	0.74–1.11	0.337
Neck length >10 mm	17 (85%)	12 (80%)	0.86	0.11–6.93	0.890
Neck angulation ≤60°	17 (85%)	15 (100%)	Inf	0.00–Inf	0.998

Data were complete. Not having the conditions served as reference group for the binary variables. Survival model: n=35; N events=13; R²=0.371; global test for proportionality of the hazards, p=0.49. Reintervention model: total n=35; N events=15; R²=0.356; global test for proportionality of the hazards, p=0.15. Bold indicates significant result.

Abbreviations: CI, confidence interval; COPD, chronic obstructive pulmonary disease; HR, hazard ratio; IFU, instructions for use; Inf, infinity.

### Development of T2EL and Aneurysm Sac Diameter

After a median of 3.4 (IQR: 0.03–85.6) months, 40% (n=14) of T2ELs sealed spontaneously. In the remaining cases (n=21), the T2EL persisted throughout the FU (Figure 1).

In 12 patients, the aneurysm sac showed shrinkage of >5 mm after the T2EL sealed (85.7%). In the persisting T2EL group, only one patient showed regression of >5 mm (4.8%; p<0.0001).

An increase in aneurysm diameter by >5 mm was only seen in the group with unsealed T2EL (14.3%, n=3, Figure 1). In one of two cases, following shortening of the proximal landing zone, a new onset of T1EL occurred due to the increase of sac diameter.

There was no change in aneurysm diameter in 19 patients (2 with sealed T2EL, 17 with persisting T2EL; Figure 1).

### Mortality

**Figure 3.**
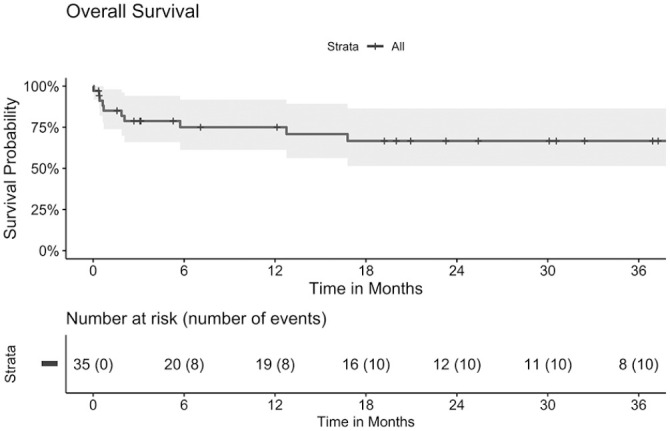
Kaplan-Meier curve of overall survival of patients with type II endoleak after endovascular treatment for ruptured abdominal aneurysm over a course of 3 years. Median (line) and 95% confidence intervals (area).

The overall survival was 85% (95% CI: 74–98%) at 30 days, 75% (95% CI: 61–92%) at 1 year, and 67% (95% CI: 51–87%) at 3 years (Figure 3).

Aneurysm-related mortality was 17.1% (n=6). Five of these patients (14%) died within the first 30 days (14.3%, Table 4). As mentioned before, one patient died due to persistent hemorrhage of T2EL at postoperative day 1. The other 4 deaths were caused by complications in the postoperative course (death on days 12 and 21 due to multiple organ failure, on day 13 due to mesenteric ischemia, and on day 19 due to pneumonia).

**Table 4.** Thirty-Day Morbidity and Mortality.

	Total (n=35) n (%)
30 day morbidity	18 (51)
Pneumonia	6 (17)
Acute renal failure/dialysis	6 (17)
Myocardial infarction	2 (6)
Mesenteric ischemia	2 (6)
Abdominal compartment syndrome	8 (23)
Persistent bleeding	2 (6)
Multi-organ failure	1 (3)
30 day mortality	5 (14)
Aneurysm-related	5 (14)
T2EL-related	1 (3)
Overall mortality	13 (37)
Aneurysm-related	6 (17)
T2EL-related	2 (6)

T2EL-related group is a subgroup of aneurysm-related group. Results are given as numbers (%).

Abbreviation: T2EL, type 2 endoleak.

During FU, one further patient died of secondary rupture due to T2EL-induced T1EL. Accordingly, T2EL-related mortality was 6% (n=2, Table 4).

Seven other deaths during FU were not aneurysm or T2EL associated. Three patients died in the context of an underlying malignancy, 2 patients died with pneumonia and respiratory insufficiency, 1 patient died with COVID-19-pneumonia. In one case, the cause of death is unknown.

The multivariable Cox model for overall survival showed that age was associated with an increased risk for mortality (HR 1.14, 95% CI: 1.04–1.26, p=0.005, Table 3). The other comorbidities were not associated with mortality. Of note, the CIs were inflated due to the low number of events and the small sample, likewise.

## Discussion

Although there is a growing number of publications on T2EL after elective EVAR, to our knowledge there is a great paucity of evidence dealing with the significance of T2EL after rAAA. This is even more astonishing since the top priority in the treatment of rAAA is the complete sealing of the aneurysm sac to avoid persistent bleeding through the rupture site. Therefore, this retrospective study investigated the short-term and midterm FU of 35 patients with T2EL after endovascular treatment of rAAA.

The overall survival with 85% at 30 days, 75% at 1 year, and 67% at 3 years was comparable to the data of rEVAR published in the literature.^19–21^ Wanhainen et al found a wide range of mortality after rEVAR from 13% to 53%, whereby in general observational studies and administrative registries mortality rates of 20% or less were described.^3^ The meta-analysis of Kontopodis et al published in 2020 found a pooled mortality after rEVAR of 25% compared with open repair of 38% within the hospital stay.^20^

Since an endovascular first strategy is strictly followed at our hospital, the 30 day reintervention rates were slightly increased as rAAA with hostile anatomy achieves significantly worse results compared with friendly anatomy.^22–24^

This also explains the increased rate of T1ELs with 11.4%, which could be identified with imaging direct postoperatively and thus be treated at an early stage. Other studies which described the outcomes after rEVAR in general had a rate of T1EL of less than 5%.^25,26^ But, on the other hand, a systematic review and meta-analysis of Kontopodis et al found no significant difference in reintervention rates between hostile and friendly anatomy.^24^

In this study group, 47% of rEVAR were performed outside the IFU. This is often accepted in emergency situations to achieve rapid bleeding control and stabilization of the patient. Similar results are described by Zarkowsky et al in a study with 621 patients. In his study, 35% of rEVAR were not performed within IFU. It is noteworthy that in-hospital mortality was significantly higher in patients treated outside the IFU.^27^ These results seem plausible, as the risk of inadequate sealing and consecutive endoleak is likely to increase with IFU noncompliance. Thus, we assessed the impact of IFU compliance on reintervention and survival in our cohort. Due to the sample size and the low number of events, the analysis inflated and instable coefficients were obtained. Nevertheless, the analysis confirmed that older age at the time of rAAA was associated with poorer overall survival. Trenner et al have already demonstrated this association in a nationwide analysis of risk factors for in-hospital mortality in patients undergoing AAA repair.^28^ Another investigation identified age older than 76 years as preoperative risk factor for mortality after repair of rAAA.^29^ Whereby Lieberg et al proved that age in elective EVAR is a predictor for 5 year mortality but not for rEVAR.^30^

The time-to-event analysis for reintervention-free survival revealed an association between arterial hypertension and the risk of reintervention (HR: 27.8, 95% CI: 1.48–521, p=0.026). The inflated CI of the HR indicates uncertainty for this estimate and hinders a conclusive interpretation.

The incidence of T2ELs in our cohort was 25%, which is comparable to the published data and is lower to the described incidence after elective EVAR.^8,9^ The incidence of T2EL in elective EVAR is described in a wide range but up to 60%.^31^ Boniakowski et al described a T2EL rate of 29% after rEVAR,^14^ whereas Quinn et al found a substantially lower incidence of T2EL in patients after rEVAR with 16% in comparison to elective EVAR with 29%.^26^ It is surprising that there is a lower rate of T2EL reported after rAAA then elective EVAR. One would expect different findings since patients after rAAA may suffer from coagulopathy hindering spontaneous occlusion of these endoleaks.^32^

Two patients showed persisting hemorrhage due to a T2EL despite successful EVAR. In total, T2EL with persistent bleeding in about 6% can be considered to be a rare complication, and has already been described in individual cases and should be therefore controlled by close postoperative hemoglobin monitoring besides the usual monitoring of vital signs.^14,15^ Both patients were treated with open surgery and ligation of the feeder vessels; one of these patients died due to a hemorrhage that could not be controlled. Another option for therapy-relevant T2EL is coil embolization of the feeder vessels, which can be a lifesaving option.^17^ However, one must take into account that endovascular therapy options in the treatment of T2EL often do not capture the entire pathology and thus not all feeder arteries of the T2EL are occluded.^7,33^ Since persistent bleeding can also lead to an ACS, an open procedure to repair the T2EL should be considered, depending on the patient’s condition. As open ligation of lumbar vessels has been reported with high success rate for elective treatment of T2EL, in our opinion and experience, it is a strategy worth considering in patients with persistent bleeding and hemodynamic instability after rEVAR.^12,34^ In patients with rAAA, this procedure enables to control the bleeding by ligation of lumbar arteries. Furthermore, decompression of the hematoma might thereby prevent or treat ACS. Thus, in many cases, it is advisable to continue with open abdomen treatment after laparotomy for ligation of lumbar arteries after rEVAR.

The possible development of an ACS should also be considered in all other endovascularly treated rAAA due to the lack of hematoma evacuation. This makes postoperative monitoring of intra-abdominal pressure essential.^35,36^

At mid term, 40% of T2ELs experienced spontaneous sealing. These patients also showed regression of the aneurysm sac in 86%. This regression has been shown in studies to be associated with better postoperative outcome. In contrast, in the remaining 60% of patients with a persisting T2EL, regression of the aneurysm sac occurred in only 5%. In 14% of these patients even an expansion of the aneurysm sac could be observed. Because the aortic wall is weakened by atrophy and proteolysis after EVAR, ruptures may well occur due to T2EL.^12^ However, the available data on this are inconsistent. While some studies report an increased risk of rupture and death, other studies show a more benign course in patients with persistent T2EL. In a recent study by Mulay et al, the benign course was reconfirmed, as the T2ELs had no effect on overall survival. Interestingly, there was also no difference between patients with reintervention for T2EL compared with untreated T2EL, emphasizing the need for conservative therapy.^37^

In our collective study, there was a secondary rupture caused by an undetected T1EL triggered by a T2EL. This must be considered because the expansion of an aneurysm sac may not only affect the diameter, but also its length. This can lead to a shortening of the landing zones and thus to a successive T1EL. Both the problem of structural wall changes and the possible growth of the aneurysm diameter and the new onset of T1EL highlighted the need for a dedicated FU program after EVAR. This is true regardless of whether the aneurysm was treated as an emergency or elective procedure.

### Limitations

Some limitations of this study must be considered. Our study is limited by its retrospective and single-center nature, as well as limited population size. A control group of patients treated with EVAR because of rAAA but without T2EL is missing. Therefore, no statistical analysis on the impact of T2EL on reintervention or survival after rEVAR was possible. Furthermore, not all endoprostheses were implanted within the IFU. This may well have an influence on the outcome, especially regarding reinterventions. However, within our limited sample, no difference in outcome was observed between patients treated inside or outside the IFU.

To our best knowledge, this study represents the largest number of patients with a T2EL after rEVAR.

## Conclusion

T2ELs after rEVAR showed a benign course in most cases. A relevant number of T2EL shows spontaneous sealing with good prognosis. In the short term, the possibility of persistent bleeding should be considered, which must be excluded by close monitoring of vital parameters and hemoglobin. In the mid term, a consequent FU protocol is required to detect known late complications after EVAR at an early stage. Especially, persistent T2EL can lead to shortening of neck, secondary T1EL, and rupture.

## References

[1] Nordon IM Hinchliffe RJ Loftus IM , et al. Pathophysiology and epidemiology of abdominal aortic aneurysms. Nat Rev Cardiol. 2011;8(2):92–102. 21079638 10.1038/nrcardio.2010.180

[2] Zhang S Feng J Li H , et al. Open surgery (OS) versus endovascular aneurysm repair (EVAR) for hemodynamically stable and unstable ruptured abdominal aortic aneurysm (rAAA). Heart Vessels. 2016;31(8):1291–1302. 26334708 10.1007/s00380-015-0736-3

[3] Wanhainen A Verzini F Van Herzeele I , et al. Editor’s choice—European Society for Vascular Surgery (ESVS) 2019 clinical practice guidelines on the management of abdominal aorto-iliac artery aneurysms. Eur J Vasc Endovasc Surg. 2019;57(1):8–93. 30528142 10.1016/j.ejvs.2018.09.020

[4] Thomas DM Hulten EA Ellis ST , et al. Open versus endovascular repair of abdominal aortic aneurysm in the elective and emergent setting in a pooled population of 37,781 patients: a systematic review and meta-analysis. ISRN Cardiol. 2014;2014:149243. 25006502 10.1155/2014/149243 PMC4004021

[5] Candell L Tucker LY Goodney P , et al. Early and delayed rupture after endovascular abdominal aortic aneurysm repair in a 10-year multicenter registry. J Vasc Surg. 2014;60(5):1146–1153. 24957409 10.1016/j.jvs.2014.05.046 PMC4331642

[6] Hobo R Buth J , EUROSTAR Collaborators. Secondary interventions following endovascular abdominal aortic aneurysm repair using current endografts. A EUROSTAR report. J Vasc Surg. 2006;43(5):896–902. 16678679 10.1016/j.jvs.2006.01.010

[7] Menges AL Trenner M Radu O , et al. Lack of durability after transarterial ethylene-vinyl alcohol copolymer-embolization of type II endoleak following endovascular abdominal aortic aneurysm repair. Vasa. 2020;49(6):483–491. 33103625 10.1024/0301-1526/a000905

[8] Gelfand DV White GH Wilson SE. Clinical significance of type II endoleak after endovascular repair of abdominal aortic aneurysm. Ann Vasc Surg. 2006;20(1):69–74. 16378143 10.1007/s10016-005-9382-z

[9] Choke E Thompson M. Endoleak after endovascular aneurysm repair: current concepts. J Cardiovasc Surg (Torino). 2004;45(4):349–366. 15365516

[10] D’Oria M Mastrorilli D Ziani B . Natural history, diagnosis, and management of type II endoleaks after endovascular aortic repair: review and update. Ann Vasc Surg. 2020;62:420–431. 31376537 10.1016/j.avsg.2019.04.048

[11] Ajalat M Williams R Wilson SE. The natural history of type 2 endoleaks after endovascular aneurysm repair justifies conservative management. Vascular. 2018;26(5):524–530. 29566590 10.1177/1708538118766103

[12] Menges AL Busch A Reutersberg B , et al. The structural atrophy of the aneurysm wall in secondary expanding aortic aneurysms with endoleak type II. J Vasc Surg. 2019;70(4):1318–1326. 30792063 10.1016/j.jvs.2018.10.091

[13] Chaikof EL Dalman RL Eskandari MK , et al. The Society for Vascular Surgery practice guidelines on the care of patients with an abdominal aortic aneurysm. J Vasc Surg. 2018;67(1):2–77.e2. 10.1016/j.jvs.2017.10.044 29268916

[14] Boniakowski AE De Martino RR Coleman DM , et al. The natural history of type II endoleaks after endovascular aneurysm repair for ruptured abdominal aortic aneurysm. J Vasc Surg. 2016;64(6):1645–1651. 27871492 10.1016/j.jvs.2016.04.063

[15] Hartung O Vidal V Marani I , et al. Treatment of an early type II endoleak causing hemorrhage after endovascular aneurysm repair for ruptured abdominal aortic aneurysm. J Vasc Surg. 2007;45(5):1062–1065. 17466801 10.1016/j.jvs.2007.01.021

[16] Koike Y Nishimura J Hase S , et al. Sac angiography and glue embolization in emergency endovascular aneurysm repair for ruptured abdominal aortic aneurysm. Cardiovasc Intervent Radiol. 2015;38(2):457–462. 24627162 10.1007/s00270-014-0873-6

[17] Ogawa Y Nishimaki H Chiba K , et al. Life-saving embolization in a patient with recurrent shock due to a type II endoleak after endovascular aortic repair for a ruptured abdominal aortic aneurysm. Ann Vasc Dis. 2015;8(2):131–134. 26131038 10.3400/avd.cr.15-00015 PMC4485037

[18] von Allmen RS Weiss S Tevaearai HT , et al. Completeness of follow-up determines validity of study findings: results of a prospective repeated measures cohort study. PLoS One. 2015;10(10):e0140817. 10.1371/journal.pone.0140817 PMC4607456 26469346

[19] Wang LJ Locham S Al-Nouri O , et al. Endovascular repair of ruptured abdominal aortic aneurysm is superior to open repair: propensity-matched analysis in the Vascular Quality Initiative. J Vasc Surg. 2020;72(2):498–507. 32273221 10.1016/j.jvs.2019.11.063

[20] Kontopodis N Galanakis N Antoniou SA , et al. Meta-analysis and meta-regression analysis of outcomes of endovascular and open repair for ruptured abdominal aortic aneurysm. Eur J Vasc Endovasc Surg. 2020;59(3):399–410. 31932143 10.1016/j.ejvs.2019.12.023

[21] Ten Bosch JA Teijink JA Willigendael EM , et al. Endovascular aneurysm repair is superior to open surgery for ruptured abdominal aortic aneurysms in EVAR-suitable patients. J Vasc Surg. 2010;52(1):13–18. 20471775 10.1016/j.jvs.2010.02.014

[22] Papazoglou K Mallios A Buster B , et al. Endovascular repair of ruptured abdominal aortic aneurysms with the endurant stent-graft: a combined experience from three centers. J Cardiovasc Surg (Torino). 2017;58(5):643–649. 10.23736/S0021-9509.16.08661-4 25996842

[23] Kansal V Nagpal S Jetty P. The effect of endograft device on patient outcomes in endovascular repair of ruptured abdominal aortic aneurysms. Vascular. 2017;25(6):657–665. 28566059 10.1177/1708538117711348

[24] Kontopodis N Tavlas E Ioannou CV , et al. Systematic review and meta-analysis of outcomes of open and endovascular repair of ruptured abdominal aortic aneurysm in patients with hostile vs. Eur J Vasc Endovasc Surg. 2020;59(5):717–728. 31948911 10.1016/j.ejvs.2019.12.024

[25] Powell JT Sweeting MJ Ulug P , et al. Editor’s choice—re-interventions after repair of ruptured abdominal aortic aneurysm: a report from the IMPROVE randomised trial. Eur J Vasc Endovasc Surg. 2018;55(5):625–632. 29503083 10.1016/j.ejvs.2018.01.028 PMC5967970

[26] Quinn AA Mehta M Teymouri MJ , et al. The incidence and fate of endoleaks vary between ruptured and elective endovascular abdominal aortic aneurysm repair. J Vasc Surg. 2017;65(6):1617–1624. 28268109 10.1016/j.jvs.2016.10.092

[27] Zarkowsky DS Sorber R Ramirez JL , et al. Aortic neck IFU violations during EVAR for ruptured infrarenal aortic aneurysms are associated with increased in-hospital mortality. Ann Vasc Surg. 2021;75:12–21. 33951521 10.1016/j.avsg.2021.04.019 PMC9843606

[28] Trenner M Kuehnl A Reutersberg B , et al. Nationwide analysis of risk factors for in-hospital mortality in patients undergoing abdominal aortic aneurysm repair. Br J Surg. 2018;105(4):379–387. 29417985 10.1002/bjs.10714

[29] Garland BT Danaher PJ Desikan S , et al. Preoperative risk score for the prediction of mortality after repair of ruptured abdominal aortic aneurysms. J Vasc Surg. 2018;68(4):991–997. 29753581 10.1016/j.jvs.2017.12.075

[30] Lieberg J Pruks LL Kals M , et al. Mortality after elective and ruptured abdominal aortic aneurysm surgical repair: 12-year single-center experience of Estonia. Scand J Surg. 2018;107(2):152–157. 29117792 10.1177/1457496917738923

[31] Alexander HC Nguyen CH Bartlett AS , et al. Reporting of clinical outcomes after endovascular aortic aneurysm repair: a systematic review. Ann Vasc Surg. 2021;77:306–314. 34437976 10.1016/j.avsg.2021.06.006

[32] Adam DJ Haggart PC Ludlam CA , et al. Coagulopathy and hyperfibrinolysis in ruptured abdominal aortic aneurysm repair. Ann Vasc Surg. 2004;18(5):572–577. 15534737 10.1007/s10016-004-0087-5

[33] Ultee KHJ BÃ¼ttner S Huurman R , et al. Editor’s choice—systematic review and meta-analysis of the outcome of treatment for type II endoleak following endovascular aneurysm repair. Eur J Vasc Endovasc Surg. 2018;56(6):794–807. 30104089 10.1016/j.ejvs.2018.06.009

[34] Yamada M Takahashi H Tauchi Y , et al. Open surgical repair can be one option for the treatment of persistent type II endoleak after EVAR. Ann Vasc Dis. 2015;8(3):210–214. 26421069 10.3400/avd.oa.14-00133 PMC4575332

[35] Rubenstein C Bietz G Davenport DL , et al. Abdominal compartment syndrome associated with endovascular and open repair of ruptured abdominal aortic aneurysms. J Vasc Surg. 2015;61(3):648–654. 25499708 10.1016/j.jvs.2014.10.011

[36] Ersryd S Djavani Gidlund K Wanhainen A , et al. Editor’s choice—abdominal compartment syndrome after surgery for abdominal aortic aneurysm: subgroups, risk factors, and outcome. Eur J Vasc Endovasc Surg. 2019;58(5):671–679. 31405726 10.1016/j.ejvs.2019.04.007

[37] Mulay S Geraedts ACM Koelemay MJW , et al. Type 2 endoleak with or without intervention and survival after endovascular aneurysm repair. Eur J Vasc Endovasc Surg. 2021;61(5):779–786. 33632609 10.1016/j.ejvs.2021.01.017

